# Daucosterol Alleviates Alcohol−Induced Hepatic Injury and Inflammation through P38/NF−κB/NLRP3 Inflammasome Pathway

**DOI:** 10.3390/nu15010223

**Published:** 2023-01-01

**Authors:** Feng Zhang, Mengyao Wang, Yang Zha, Jie Zhou, Jihong Han, Shuang Zhang

**Affiliations:** Key Laboratory of Metabolism and Regulation for Major Diseases of Anhui Higher Education Institutes, College of Food and Biological Engineering, Hefei University of Technology, Hefei 230002, China

**Keywords:** Daucosterol, alcoholic liver disease, p38, NF−κB, NLRP3 inflammasome

## Abstract

Alcoholic liver disease (ALD) is caused by chronic excessive alcohol consumption, which leads to inflammation, oxidative stress, lipid accumulation, liver fibrosis/cirrhosis, and even liver cancer. However, there are currently no effective drugs for ALD. Herein, we report that a natural phytosterol Daucosterol (DAU) can effectively protect against liver injury caused by alcohol, which plays anti−inflammatory and antioxidative roles in many chronic inflammatory diseases. Our results demonstrate that DAU ameliorates liver inflammation induced by alcohol through p38/nuclear factor kappa B (NF−κB)/NOD−like receptor protein−3 (NLRP3) inflammasome pathway. Briefly, DAU decreases NF−κB nuclear translocation and inhibits NLRP3 activation by decreasing p38 phosphorylation. At the same time, DAU also protects against hepatic oxidative stress and lipid accumulation. In conclusion, our research provides a new clue about the protective effects of naturally active substances on ALD.

## 1. Introduction

Alcoholic liver disease (ALD) is a common injury to the liver caused by excessive alcohol consumption [1]. It has been reported that alcohol was responsible for 3% of all global deaths, highlighting the importance of ALD treatment in 2020 [2]. Long−term excessive drinking could lead to hepatic injury including liver steatosis, hepatitis and liver fibrosis/cirrhosis, and more seriously, hepatocellular carcinoma [3,4].

Alcohol has been recognized as a direct hepatic toxin in recent decades. The liver is the central organ of alcohol metabolism. Over 95% of alcohol ingestion is metabolized in the liver, and the remainder is excreted directly by urine, sweat, and breathing [5]. When ethanol is metabolized in vivo, it is first metabolized into acetaldehyde by alcohol dehydrogenase (ADH), and cytochrome P450 family two subfamily E member 1 (CYP2E1), as a key enzyme in ethanol metabolism, also participates in this process [6,7]. During this period, many reactive oxygen species (ROS) will be produced, which causes oxidative stress and catalyzes lipid peroxidation [8,9]. High levels of ROS also aggravate inflammation and activate NF−κB and mitogen−activated protein kinases (MAPKs) proinflammatory pathways, promoting the production of inflammatory factors, like interleukin (IL)−1β, IL−6 and tumor necrosis factor (TNF)−α. Then, aldehyde dehydrogenase 2 (ALDH2) metabolizes acetaldehyde into acetic acid [10]. At present, drugs that can be used to treat alcoholic liver disease include corticosteroids and pentoxifylline, but the therapeutic effect is not significant [11]. Therefore, it is promising to look for natural and effective ingredients to alleviate ALD.

Daucosterol (DAU) is a phytosterol glycoside widely existing in plants, which has been successively used in the study of pharmacological action and disease prevention after isolation [12,13]. Bui et al. extracted DAU from *Sanchezia speciosa*, and found that it had a certain antioxidative capacity, and the half inhibitory concentration (IC50) was 82.50 μg/L determined by 2,2−diphenyl−1−hydrazyl (DPPH) method [14]. Chung et al. found that DAU downregulates the MAPK pathway to provide neuroprotection and upregulating heme oxygenase−1 (HO−1), catalase (CAT), and superoxide dismutase (SOD) 2 antioxidative genes and is associated with reduced oxidative stress in SK−N−SH cells [15]. In addition, DAU can also protect against liver fibrosis induced by CCL4 [16].

However, DAU’s role in ALD remains unclear. Here, we are devoted to exploring the beneficial effect of DAU on ALD and clarifying its potential mechanism. We found that DAU could alleviate alcohol−induced inflammation, oxidative stress, and lipid accumulation by regulating p38/NF−κB/NLRP3 inflammasome pathway. Our research indicated that DAU has a very good prospect for treating ALD.

## 2. Materials and Methods

### 2.1. Reagents

Daucosterol (DAU, Cat# CSN10899) was purchased from CSNpharm (Chicago, IL, USA). Anisomycin (Cat# HY−18982) was purchased from MedChemExpress (Middlesex, NJ, USA). Picrosirius Red staining kit (Cat# G1742) was purchased from Solarbio (Beijing, China). 3−(4,5−Dimethylthiazol−2−yl)−2,5−diphenyltetrazolium bromide (MTT) (Cat# M2003) was purchased from Sigma−Aldrich (Louis, MI, USA). Rabbit anti−p38 (Cat# A14401), p−p38 (Cat# AP0526), ALDH2 (Cat# A1226), SOD1 (Cat# A0274), SOD2 (Cat# A1340), IL−1β (Cat# A20529), NLRP3 (Cat# A5652), caspase−1 (Cat# A0964), and ASC (Cat# A1170) antibodies were obtained from Abclonal (Wuhan, China). Rabbit anti−SREBP1c (Cat# 66875−1−Ig), SCD1 (Cat# 23393−1−AP), CYP2E1 (Cat# 67263−1−Ig), α−tubulin (Cat# 11224−1−AP), and β−actin (Cat# 20536−1−AP) were obtained from Proteintech Group (Chicago, IL, USA). Rabbit anti−COL3A1 (Cat# AF5457), Nrf2 (Cat# AF0639), p−NF−κB (Cat# AF0639), NF−κB (Cat# AF5006), and α−SMA (Cat# AF1032) antibodies were purchased from Affinity Biosciences, Inc. (Cincinnati, USA). Mouse anti−FASN (Cat# sc−55580) and COL1A1 (Cat# sc−59772) antibodies Goat anti−rabbit IgG (H+L)−HRP (Cat# LK2001) and goat anti−mouse IgG (H+L)−HRP (Cat# LK2003) antibodies were purchased from Sungene Biotech (Tianjin, China). Rhodamine−conjugated goat anti−rabbit IgG (H+L) and FITC−conjugated goat anti−rabbit IgG (H+L) were obtained from Proteintech (Chicago, IL, USA).

### 2.2. Experimental Animals

The 8−week−old male C57BL/6J mice were provided by GemPharmatech (Nanjing, China). During the experiment, all mice were kept in a light/dark cycle of 12 h at 22–24 °C and were given unlimited access to food and water.

### 2.3. Design of Animal Experiment

**Figure 1:**
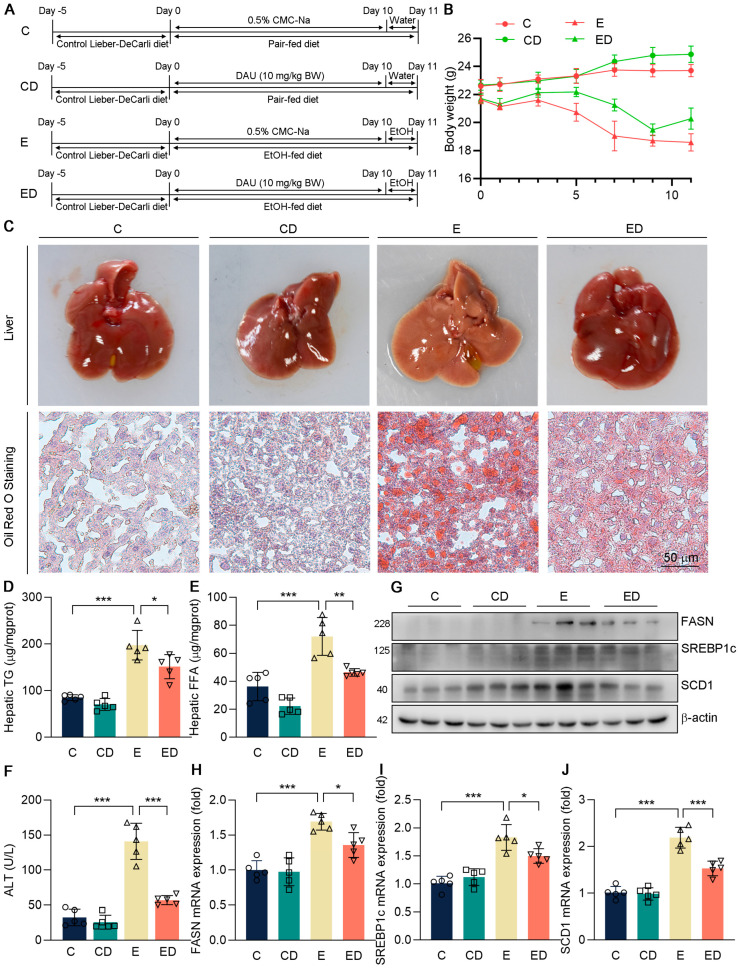
The effect of DAU on hepatic lipid accumulation and liver injury induced by alcohol. (**A**) Schematic diagram of animal experiments. (**B**) Weight change of mice. (**C**) Liver and hepatic Oil Red O staining of mice. Hepatic (**D**) TG and (**E**) FFA levels. (**F**) Serum ALT level. (**G**–**J**) Protein and mRNA expression of FASN, SREBP1c, and SCD1 in mice liver. Data are mean ± SD using one−way ANOVA (*n* ≥ 3), * *p* < 0.05, ** *p* < 0.01, *** *p* < 0.001.

Mice were treated with a short−term chronic (10 days)−plus−binge ethanol feeding model to induce ALD, according to the National Institute on Alcohol Abuse and Alcoholism (NIAAA) protocol [17]. Firstly, the Lieber−DeCarli diet was given to all mice for 5 days to acclimate to a lipid diet. After that, mice were provided sustained feeding with the Lieber−DeCarli diet containing 5% (vol/vol) ethanol for 10 days. 9 h before euthanizing, the mice were given ethanol (5 g/kg body weight) intragastrically. Control groups were fed a control diet with the same calories. Four groups of mice were set up (*n* = 8 in each group) in our experiment: control group (C), DAU group (CD), EtOH group (E), and EtOH plus DAU group (ED) (Figure 1A). The mice in CD and ED were administered intragastrically with DAU (10 mg/kg body weight) for 10 days.

### 2.4. Biochemical Analysis

The alanine transaminase (ALT) in serum was tested by an automatic biochemical analyzer [18]. The hepatic SOD, glutathione (GSH), and CAT were tested with a kit. IL−1β and TNF−α in serum were tested with ELISA kit.

### 2.5. Histopathological Analysis

Mouse liver tissue was fixed in paraformaldehyde and embedded in paraffin. Sections were prepared and subjected to H&E staining, Picrosirius Red staining, and Masson staining [18,19]. Frozen liver tissue cut to 5 μm was subjected to Oil Red O staining and DHE staining [20].

### 2.6. Cell Culture

HepG2 cells were provided by ATCC (Rockville, USA) and maintained in MEM medium supplemented with 10% FBS and 50 mg/mL penicillin/streptomycin in a 5% CO_2_ incubator.

### 2.7. Cell Viability Assay

The cell viability was detected by MTT assay as previously described [21]. Briefly, after indicated treatment, MTT solution (5 mg/mL/well) was added to cells. Then, 200 μL/well of DMSO was added. The detection wavelength is 550 nm.

### 2.8. Determination of Cellular ROS Levels

Briefly, after treatment, 5 mM DCFH−DA solution (30 μL/well) was added to cells. Then cells were washed, followed by the determination of fluorescence at 488 nm (excitation) and 525 nm (emission) [21].

### 2.9. Immunofluorescence Cytochemistry

Cells were fixed and then permeabilized with 0.5% Triton X−100. After rinsing, the cells were blocked and incubated with antibodies against NF−κB and Nrf2 overnight. After washing, the cells were incubated with secondary antibodies and then counterstained with DAPI [19].

### 2.10. Western Blotting

Western blotting was carried out as described [20]. In brief, samples were lysed with a lysis buffer. The same quantity of total proteins (40–60 μg) from each sample was separated on SDS−PAGE followed by transfer onto a nitrocellulose filter membrane and incubation with the indicated antibodies. After incubation with secondary antibodies, the protein bands were visualized using a chemiluminescence imaging system. All samples in the same group were processed simultaneously.

### 2.11. Quantitative Real−Time PCR (qRT−PCR)

Cells and liver tissues were extracted for total RNA. A reverse transcription kit was used to synthesize cDNA. qRT−PCR was performed with the primers listed in Table 1 using the SYBR green PCR master mix. The coding sequence of the target gene was found in NCBI, and Primer3 (v.0.4.0) was used to design amplification primers. The results were normalized by β−actin in the corresponding sample [19].

### 2.12. Statistical Analysis

All data confirmed a normal distribution and were presented as the mean ± standard deviation (SD). Experiments were repeated at least three times. Statistical analysis was performed using GraphPad Prism 8.0 software and analyzed using one−way analysis of variance (ANOVA) with a Tukey post−hoc test for multiple comparisons. A significant difference was considered if *p* < 0.05 (*n* ≥ 3).

## 3. Results

### 3.1. Daucosterol Alleviates Hepatic Lipid Accumulation and Liver Injury Induced by Alcohol

To determine the protective effects of DAU against ALD, C57BL/6J male mice were orally administrated with DAU in an ALD model according to Figure 1A. Briefly, mice were provided sustained feeding with the Lieber−DeCarli diet for 10 days. DAU (10 mg/kg) or 0.5% CMC−Na alone were administered intragastrically. Then, 9 h before euthanizing, the mice were given ethanol (5 g/kg body weight) orally. C groups were pair−fed on a control diet. Firstly, we determined the body weight change during the experiment. Compared with the C group, the body weight of mice taking an alcoholic diet decreased significantly (Figure 1B), which was consistent with the previous result that alcohol intake had a negative effect on the growth of mice [22]. However, the body weight change of mice supplemented with DAU was improved, indicating that DAU may have a protective effect on an alcohol diet.

Next, we determined the effects of DAU on hepatic injury induced by alcohol. As shown in Figure 1C, whitey liver color was shown in the E group compared with C and CD groups, indicating that alcohol causes hepatic steatosis in mice, while DAU treatment ameliorated hepatic steatosis. The result also exhibited that severe lipid accumulation occurred in the E group, which could be reversed by DAU (Figure 1C). To further determine the lipid profiles in the liver, the contents of TG and FFA were detected (Figure 1D,E). It showed the hepatic TG and FFA levels were significantly inhibited by DAU treatment compared with the E group. In Figure 1F, DAU significantly decreased serum ALT level compared with the E group, which indicated less liver injury. Consistently, DAU could effectively reduce the upregulation of lipid synthesis genes FASN, SREBP1c, and SCD1 induced by alcohol (Figure 1G–J and Figure S1). These results suggest that DAU alleviates alcohol−induced hepatic lipid accumulation in mice.

### 3.2. Daucosterol Alleviates Hepatic Oxidative Stress Induced by Alcohol

**Figure 2:**
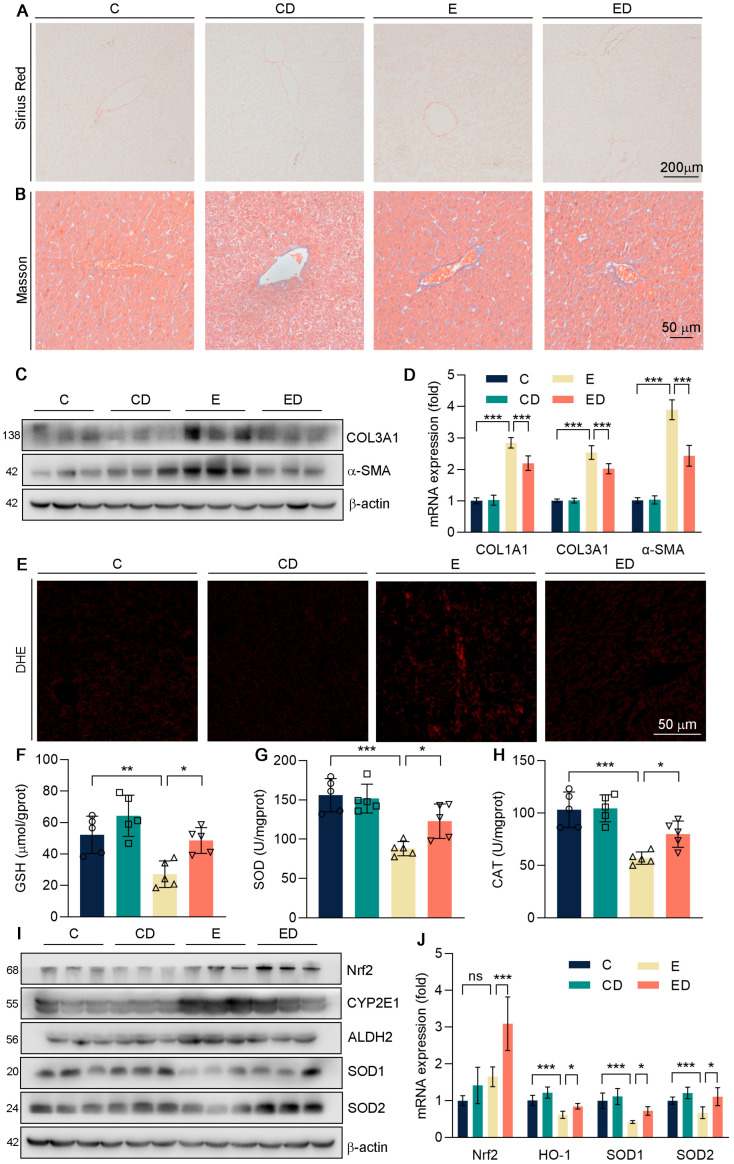
The effect of DAU on hepatic oxidative stress induced by alcohol. (**A**) Hepatic Picrosirius Red staining of mice. (**B**) Hepatic Masson staining of mice. (**C**,**D**) Protein and mRNA expression of COL1A1, COL3A1, and α−SMA in mice liver. (**E**) Hepatic DHE staining of mice. (**F**–**H**) Hepatic GSH, SOD, and CAT levels. (**I**) Protein expression of Nrf2, CYP2E1, ALDH2, SOD1, and SOD2 in mice liver. (**J**) mRNA expression of Nrf2, HO−1, SOD1, and SOD2 in mice liver. Data are mean ± SD using one−way ANOVA (*n* ≥ 3), * *p* < 0.05, ** *p* < 0.01, *** *p* < 0.001, ns: not significant.

It is well known that alcohol intake could cause liver fibrosis [23]. In Figure 2A,B, Short periods of alcohol consumption did not cause liver fibrosis. However, the marker genes of fibrosis like COL1A1, COL3A1, and α−SMA, were detected in mice liver (Figure 2C,D and Figure S1) and it showed that COL1A1, COL3A1, and α−SMA were up−regulated by alcohol and reversed by DAU. These results suggest that DAU may slow the progression of fibrosis.

Scholars have reported that when alcohol is catabolized in the body, it will produce a great deal of ROS, which will disrupt lipid metabolic homeostasis and lead to oxidative damage [24]. Therefore, we conducted DHE staining with mice liver sections. It showed that alcohol caused the accumulation of ROS in the liver, and DAU alleviated oxidative damage (Figure 2E). GSH is a non−enzymatic antioxidant in the liver that could alleviate oxidative stress [25,26]. Antioxidant enzymes SOD and CAT also play important roles in defending against oxidative stress [27]. As shown in Figure 2F–H, we found that the expression of GSH, SOD, and CAT was significantly decreased by alcohol, but DAU could enhance their expression. It showed that DAU could alleviate the oxidative stress induced by alcohol. At the same time, the levels of ALDH2 and CYP2E1 were detected in mice liver, which promoted the metabolism of alcohol in vivo, thus producing a large amount of ROS. As shown in Figure 2I,J, alcohol promotes the expression of ALDH2 and CYP2E1, but it was reversed effectively by DAU. Further, DAU upregulated Nrf2, one of the key antioxidative transcription factors (Figure 2I,J) [28,29]. Subsequently, antioxidative genes SOD1 and SOD2, downstream of Nrf2, were also increased by DAU (Figure 2I,J). The above results show that DAU had effective antioxidative ability in ALD.

### 3.3. Daucosterol Alleviates Hepatic Inflammation Induced by Alcohol and Regulates P38 Pathway

**Figure 3:**
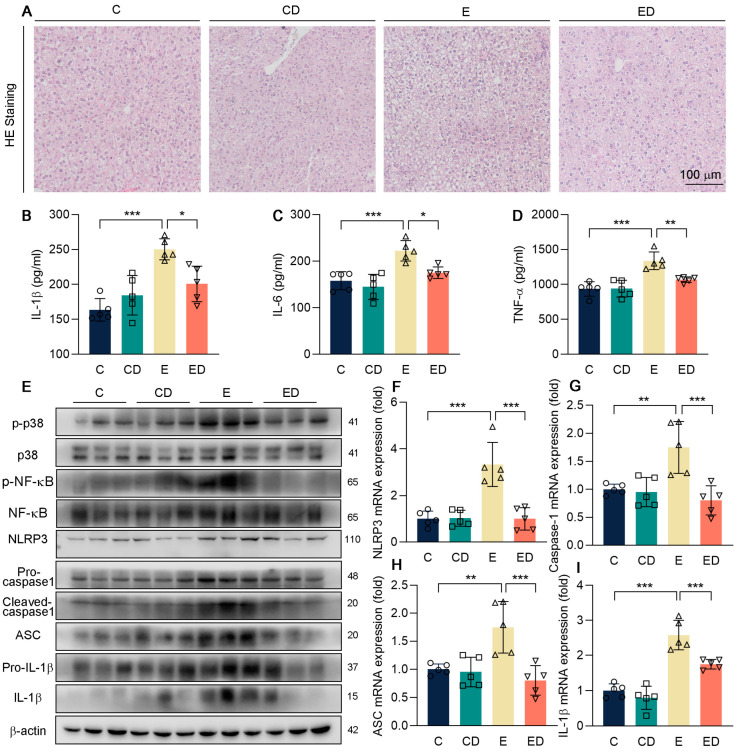
The effect of DAU on hepatic inflammation induced by alcohol. (**A**) Hepatic H&E staining of mice. (**B**–**D**) Serum IL−1β, IL−6, TNF−α levels. (**E**) Protein expression of p−p38, p38, p−NF−κB, NF−κB, NLRP3, pro−caspase1, cleaved−caspase1, ASC, pro−IL−1β and IL−1β in mice liver. (**F**–**I**) mRNA expression of NLRP3, caspase1, ASC, and IL−1β in mice liver. Data are mean ± SD using one−way ANOVA (*n* ≥ 3), * *p* < 0.05, ** *p* < 0.01, *** *p* < 0.001.

Next, to determine the effect of DAU in alcohol−induced hepatic injury, firstly we conducted H&E staining as shown in Figure 3A. It showed that compared with the C group, the E group developed severe liver injury under the treatment of alcohol. However, DAU significantly decreased serum ALT levels, which indicated less liver injury. A major cause of hepatic damage caused by alcohol is inflammation [1,30,31]. Serum proinflammatory factors IL−1β, IL−6, and TNF−αdetected by ELISA showed that DAU could inhibit hepatic inflammation induced by alcohol (Figure 3B–D).

Studies have shown that ALD can be alleviated by regulating the p38 pathway [32]. To further explore the mechanism of DAU in ameliorating liver inflammation, phosphorylation of p38 and NF−κB, and the activation of NLRP3 inflammasome were detected (Figure 3E–I and Figure S3). It showed that DAU decreased the phosphorylation levels of p38 and NF−κB in contrast to the E group. At the same time, the expression of NLRP3, cleaved−caspase−1, ASC, and IL−1β was also downregulated by DAU. The above results suggest that DAU may ameliorate alcohol−induced hepatic inflammation by regulating inflammasome activation through p38/NF−κB pathways.

### 3.4. DAU Alleviates Genes Expression of Lipid Synthesis in HepG2 Cells Induced by Alcohol

The in vivo studies above in mice have proved that DAU could relieve alcohol-induced hepatic injury. Next, HepG2 cells were applied to further verify the effect and explore the potential mechanism of DAU in vitro.

**Figure 4:**
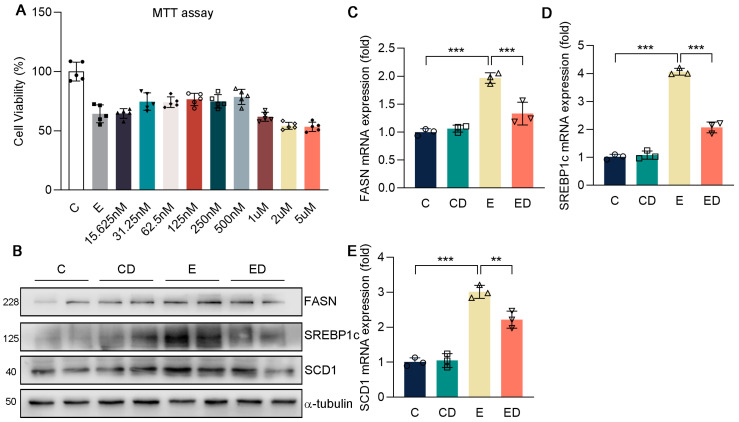
The effect of DAU on gene expression of lipid synthesis in HepG2 cells induced by alcohol. (**A**) MTT assay. (**B**–**E**) Protein and mRNA expression of FASN, SREBP1c, SCD1, COL1A1, COL3A1, and α−SMA in HepG2. Data are mean ± SD using one−way ANOVA (*n* ≥ 3), ** *p* < 0.01, *** *p* < 0.001.

First of all, the appropriate concentration of DAU on HepG2 in vitro was determined by MTT assay (Figure 4A). It showed that alcohol inhibited cell viability, while the DAU of 31.25–500 nM could slightly alleviate the decrease of cell viability caused by alcohol. We chose the concentration with 500 nM of DAU for the subsequent experiments. Previous in vivo studies have shown that DAU can alleviate liver fibrosis and lipid accumulation in mice liver. Therefore, HepG2 cells were applied for verification in vitro, and the results were consistent with the results in vivo. Briefly, alcohol promoted FASN, SREBP1c, and SCD1 expression in lipid synthesis. However, after DAU treatment, these changes were reversed (Figure 4B–E and Figure S4). Combined with the in vivo and in vitro study, we confirmed that DAU can alleviate liver lipid accumulation.

### 3.5. DAU Alleviates Oxidative Stress in HepG2 Cells Induced by Alcohol

**Figure 5:**
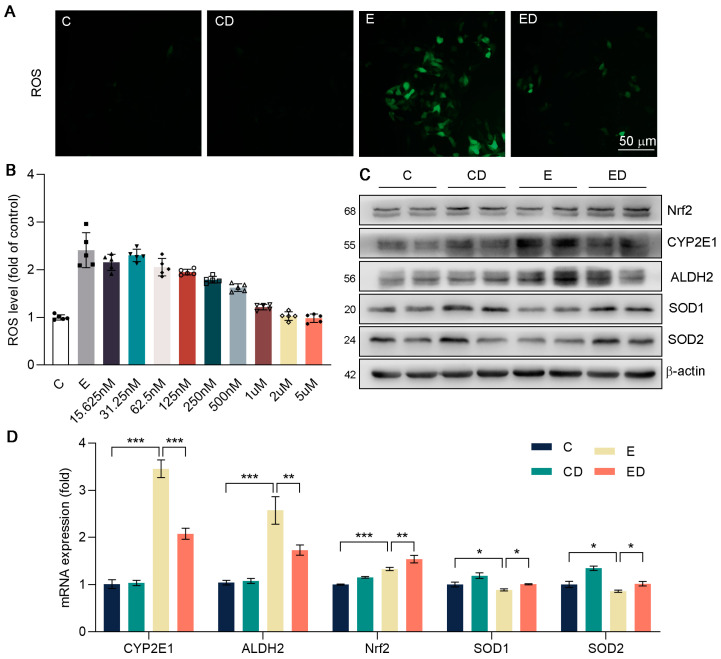
**The** effect of DAU on oxidative stress in HepG2 cells induced by alcohol. (**A**) DCFH−DA staining of HepG2. (**B**) ROS levels of HepG2. (**C**) Protein expression of Nrf2, CYP2E1, ALDH2, SOD1 and SOD2 in HepG2. (**D**) mRNA expression of Nrf2, CYP2E1, ALDH2, SOD1 and SOD2 in HepG2. Data are mean ± SD using one−way ANOVA (*n* ≥ 3), * *p* < 0.05, ** *p* < 0.01, *** *p* < 0.001.

Next, we conducted DCFH−DA staining in HepG2. In Figure 5A, DAU had an obvious inhibitory effect on oxidative stress caused by alcohol. In addition, in Figure 5B, the ROS levels of HepG2 were detected under the same concentration of alcohol and DAU with MTT assay. We chose 500 nM of DAU for the subsequent experiments. It showed that ALDH2 and CYP2E1 expression were increased in HepG2 due to alcohol and were downregulated by DAU (Figure 5C and Figure S5). In addition, compared with the E group, the expression of antioxidative genes in the ED group was significantly increased by DAU (Figure 5D). These results suggest that DAU can alleviate the rate of alcohol metabolism and avoid acute oxidative injury caused by excessive alcohol metabolism.

### 3.6. DAU Alleviates Alcohol−Induced Inflammation in HepG2 Cells by Regulating P38/NF−κB/NLRP3 Inflammasome Pathway

**Figure 6:**
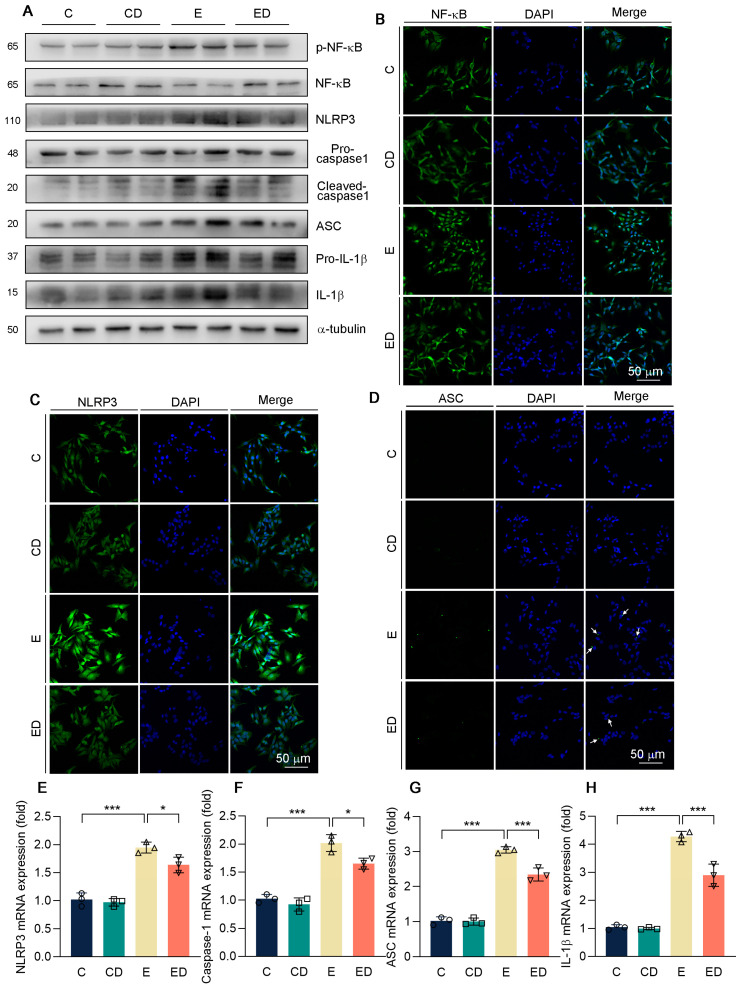
The effect of DAU on oxidative stress in HepG2 cells induced by alcohol. (**A**) Protein expression of p−NF−κB, NF−κB, NLRP3, pro−caspase−1, cleaved−caspase1, ASC, pro−IL−1β and IL−1β in HepG2. (**B**–**D**) Immunofluorescent staining of NF−κB, NLRP3, and ASC in HepG2. (**E**–**H**) mRNA expression of NLRP3, caspase−1, ASC, and IL−1β in HepG2 cells. Data are mean ± SD using one−way ANOVA (*n* ≥ 3), * *p* < 0.05, *** *p* < 0.001.

Consistent with the in vivo results, it was suggested that alcohol promoted NF−κB phosphorylation and NLRP3 inflammasome activation, and further promoted IL−1β release (Figure 6A and Figure S1). As expected, DAU attenuated alcohol−induced inflammation. Consistently, immunofluorescence images exhibited that obvious nuclear translocation occurred of NF−κB, and alcohol upregulated NLRP3 and ACS in the E group compared with the C group, but DAU reversed this trend (Figure 6B–D). Similarly, the expression of NLRP3 inflammasome−related genes and IL−1β were inhibited by DAU in alcohol−treated HepG2 cells (Figure 6E–H). These indicated that the improvement of alcohol−induced inflammation by DAU is associated with the regulation of NF−κB and NLRP3 inflammasome.

**Figure 7:**
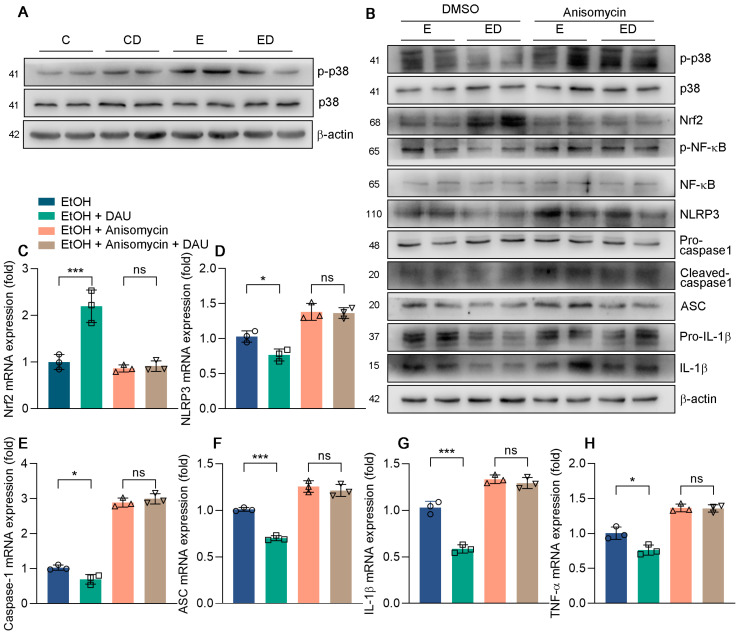
The effect of anisomycin in DAU ameliorated liver inflammation induced by alcohol. (**A**,**B**) Protein expression of p−p38, p38, p−STAT3, STAT3, p−NF−κB, NF−κB, NLRP3, pro−caspase−1, cleaved−caspase1, ASC, pro−IL−1β and IL−1β in HepG2. (**C**–**H**) mRNA expression of Nrf2, NLRP3, caspase−1, ASC, IL−1β and TNF−α in HepG2. Data are mean ± SD using one−way ANOVA (*n* ≥ 3), * *p* < 0.05, *** *p* < 0.001, ns: not significant.

Previous results indicated that DAU could regulate the phosphorylation of p38 in mice, which was also verified in HepG2 cells [33]. Compared with the E group, DAU had a significant effect on decreasing p−p38 (Figure 7A and Figure S7). Studies have shown that the p38 pathway could reduce acute lung injury in mice by regulating NLRP3 inflammasome [34,35]. Therefore, we tested whether DAU regulates NLRP3 inflammasome activation through the p38 pathway. Anisomycin is a p38 agonist that promotes p38 phosphorylation. As shown in Figure 7B and Figure S8, the inhibition of p38 phosphorylation by DAU was ineffective after anisomycin treatment. At the same time, the increase of Nrf2 and the decrease of NF−κB phosphorylation by DAU were also reversed by anisomycin treatment. Subsequently, DAU also lost its effect on NLRP3, caspase−1, and ASC, which in turn affected the release of inflammatory factors (Figure 7B–H). The results show that the anti−oxidative and anti−inflammatory effects of DAU against alcoholic liver disease are dependent on p38 activation and can be reversed by p38 agonist anisomycin treatment. In conclusion, we determine that DAU alleviates hepatic injury and inflammation induced by alcohol by regulating the p38/NF−κB/NLRP3 inflammasome pathway.

## 4. Discussion

ALD accounts for a large proportion of worldwide liver disease. The liver is the central organ for alcohol metabolism. Alcohol can cause hepatic oxidative stress, inflammation, steatosis, fibrosis, and even hepatocellular carcinoma [36]. However, there is currently no drug for effectively treating ALD. Many studies have shown that a large amount of natural bioactive substances, such as quercetin, fisetin, anthocyanins, have protective effects on ALD [37,38,39]. DAU is a natural phytosterol, which exerts its biological activity in many diseases and has many effects such as relieving inflammation and oxidative stress [12,40,41]. It is worth studying the effects of DAU in ALD and revealing the underlying mechanism. 

The metabolic process of ethanol generating into acetate in vivo is mainly participated by ADH and ALDH [42]. ADH firstly oxidizes ethanol to acetaldehyde, and then acetaldehyde is further oxidized to acetate by ALDH. CYP2E1 is also an important part of alcohol metabolism. In this process, covalent chemical adducts can be formed when acetaldehyde combines with proteins, lipids, and DNA [43,44]. These adducts could alter cellular homeostasis, and promote DNA damage and mutation. Under normal circumstances, only a little ethanol is oxidized to acetaldehyde by CYP2E1 [45]. However, during alcoholism, the expression of CYP2E1 increases, thereby generating a large number of ROS and causing severe liver damage [46]. In our research, we showed that DAU reduces the expression of CYP2E1 and ALDH2, thereby avoiding the large amount of oxidative stress generated by alcohol metabolism within a short time.

The earliest reaction of alcohol abuse is the accumulation of lipids in hepatocytes [24]. N Grunnet et al. declared that ethanol slows lipid β−oxidation and accelerates fatty acid intake [47]. Peroxisome proliferator−activated receptor α (PPARα) regulates the transcription of esterification genes and fatty acids excretion. Studies also showed that the inhibition of PPARα activity by ethanol is associated with lipid accumulation [48]. In addition, ethanol up−regulates CYP2E1, resulting in increased oxidative stress, which can also inhibit PPARα [49]. Mengyao Hu et al. determined D−mannose regulates abnormal lipid metabolism in ALD by inhibiting the expression of SREBP1c, ACC1, and FASN [50]. In our experiment, DAU reduced the increase of hepatic TG and FFA induced by alcohol and significantly inhibited the lipid synthesis gene SREBP1c, FASN, and SCD1. The liver will gradually develop fibrosis in the case of long−term alcoholism, which is characterized by the increase of fibrillar collagens type I and type III [51]. DAU reduced COL1A1, COL3A1, and α−SMA expression, which helped alleviate fibrosis.

Oxidative stress is important in the development of ALD. A great deal of ROS is produced in alcohol metabolism. Usually, antioxidants such as SOD, CAT, and GSH can remove excessive ROS to ensure balance in vivo, but excessive alcohol intake will disrupt this balance [9]. In our research, the protective effects of DAU on liver SOD, CAT, and GSH were evaluated. In addition, Jang J et al. declared that DAU reduces ROS induced by dextran sulfate sodium [52]. Our results indicated that DAU recovered the decrease of antioxidative factors induced by alcohol, and DHE staining also showed that ROS decreased significantly under the treatment of DAU. 

The accumulation of ROS in liver caused by ethanol can also lead to inflammation. Several studies have shown that NLRP3 inflammasome plays a vital role in inflammatory diseases. Petrasek et al. first demonstrated that IL−1β activation in ALD is associated with inflammasome [53]. Zhou et al. found that alcohol promotes IL−1β and IL−18 expression through NLRP3 inflammasome activation [30]. At the same time, alcohol also promotes the nuclear translocation of NF−κB, then inflammation is activated. Our research showed that DAU inhibits the activation of NLRP3 and decreases the phosphorylation of NF−κB, thus playing an anti−inflammatory role.

MAPKs participate in the regulation of various cellular processes. The p38 MAPK is an important member of MAPKs, which not only involves the cell cycle, cell death, development, differentiation, senescence, and tumorigenesis but also regulates inflammation as a specific serine/threonine kinase [35]. Research has shown that inhibition of p38 plays a protective role in many inflammatory disease models, including Parkinson’s disease, acute lung injury, colitis, and diabetic nephropathy, by regulating the activation of NLRP3 inflammasome [54,55,56,57]. In our study, p38 was phosphorylated by alcohol and inhibited by DAU in the ALD mice model. Therefore, we speculated whether DAU attenuated NLRP3 activation through the p38 pathway. Anisomycin was used to activate the p38 pathway to verify the underlying anti−inflammatory mechanism of DAU. The in vitro results showed that DAU lost its inhibitory effect on NF−κB and NLRP3 inflammasome activation in the presence of anisomycin, indicating that p38 plays a key role in this process.

Here, we investigated the effects of DAU on ALD and briefly explored its underlying mechanism. Fortunately, DAU could alleviate alcohol−induced liver inflammation induced by regulating p38/NF−κB/NLRP3 pathway, and it also has significant effects on hepatic steatosis and oxidative stress in the liver. Our results suggested that the phosphorylation of signal transducer and activator of transcription (STAT) 3 was also regulated by DAU (Figure S9). Furthermore, STAT3 plays a key role in liver disease pathogenesis [58]. Many natural products that inhibit the activation of STAT3 have significant inhibitory effects on markers of liver fibrosis [59,60,61]. The ability of DAU to improve liver fibrosis cannot be effectively tested due to the limitations of the model. However, the effect of DAU on the activation of STAT3 showed that it has the potential to improve liver fibrosis. Katharina et al. demonstrated that the downregulation of STAT3 was also associated with anti−inflammatory effects in an inflammation model in HepG2 cells [62]. Much evidence shows that STAT3 is closely related to ALD. Therefore, detailed studies should be conducted in the future to reveal the role of STAT3 in DAU−ameliorated ALD. In conclusion, we provide a new clue for the protection of ALD by natural active substances.

## Figures and Tables

**Table 1:** Sequences of qRT−PCR primers.

Gene	Forward	Backward
FASN	CACGCATATACCCGCTACCT	CCAGAGTGTTCATTCGAGCA
SREBP1c	GGATTGCACTTTCGAAGACATG	AGCATAGGGTGGGTCAAATAGG
SCD1	CCCAGCTGTCAAAGAGAAGG	CAAGAAAGTGGCAACGAACA
COL1A1	GACGCCATCAAGGTCTACTG	ACGGGAATCCATCGGTCA
COL3A1	CAAGAAAGTGGCAACGAACA	ATCCATCTTTGCCATCTTCG
α−SMA	ACTGGGACGACATGGAAAAG	GTTCAGTGGTGCCTCTGTCA
Nrf2	TCACACGAGATGAGCTTAGGGCAA	TACAGTTCTGGGCGGCGACTTTAT
HO−1	CACGCATATACCCGCTACCT	CCAGAGTGTTCATTCGAGCA
SOD1	CCAGTGCAGGACCTCATTTT	TCATGGACCACCATTGTACG
SOD2	TCAATGGTGGGGGACATATT	GAACCTTGGACTCCCACAGA
NLRP3	AAAGGAAGTGGACTGCGAGA	CCCTTTTCGAATTTGCCATA
caspase−1	CTCAGGCTCAGAAGGGAATG	CGCTGTACCCCAGATTTTGT
ASC	TCTGTACGGGAAGGTCCTGA	TCCTCCACCAGGTAGGACTG
IL−1β	GACCTTCCAGGATGAGGACA	AGCTCATATGGGTCCGACAG
CYP2E1	ACCCGAGACACCATTTTCAG	TCCAGCACACACTCGTTTTC
ALDH2	ACAATGGCAAGCCCTATGTC	ACAGGTTCATGGCGTGTGTA
TNF−α	CGTCGTAGCAAACCACCAAG	TTGAAGAGAACCTGGGAGTAGACA
β−actin	ATGGAGGGGAATACAGCCC	TTCTTTGCAGCTCCTTCGTT

## Data Availability

Not applicable.

## Supplementary Materials

The following supporting information can be downloaded at: https://www.mdpi.com/article/10.3390/nu15010223/s1, Figure S1: Quantitative analysis of protein in Figure 1A; Figure S2: Quantitative analysis of protein in Figure 2C; Figure S3: Quantitative analysis of protein in Figure 3E; Figure S4 Quantitative analysis of protein in Figure 4B; Figure S5: Quantitative analysis of protein in Figure 5C; Figure S6: Quantitative analysis of protein in Figure 6A; Figure S7: Quantitative analysis of protein in Figure 7A; Figure S8: Quantitative analysis of protein in Figure 7B; Figure S9: Protein expression of p−STAT3 and STAT3 in HepG2 cells induced by alcohol.
