# Supplementary material for: Daucosterol Alleviates Alcohol−Induced Hepatic Injury and Inflammation through P38/NF−κB/NLRP3 Inflammasome Pathway
Source: Nutrients. 2023 Jan 1;15(1):223. doi: 10.3390/nu15010223 (PMC9823995; doi:10.3390/nu15010223)
Supplement: Supplementary file 1 [file nutrients-15-00223-s001.zip › nutrients-2088339-supplementary/supplementary information.pdf]

For all symbols, * $p < 0.05$, ** $p < 0.01$, *** $p < 0.001$

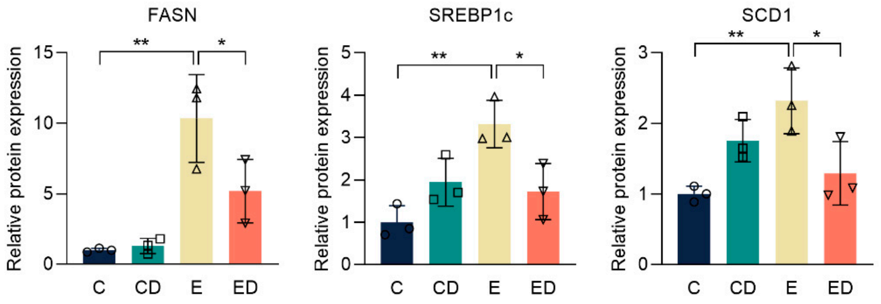

Figure S1. Quantitative analysis of protein in Figure 1A.

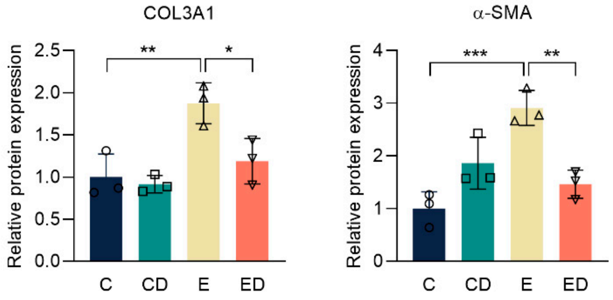

Figure S2. Quantitative analysis of protein in Figure 2C.

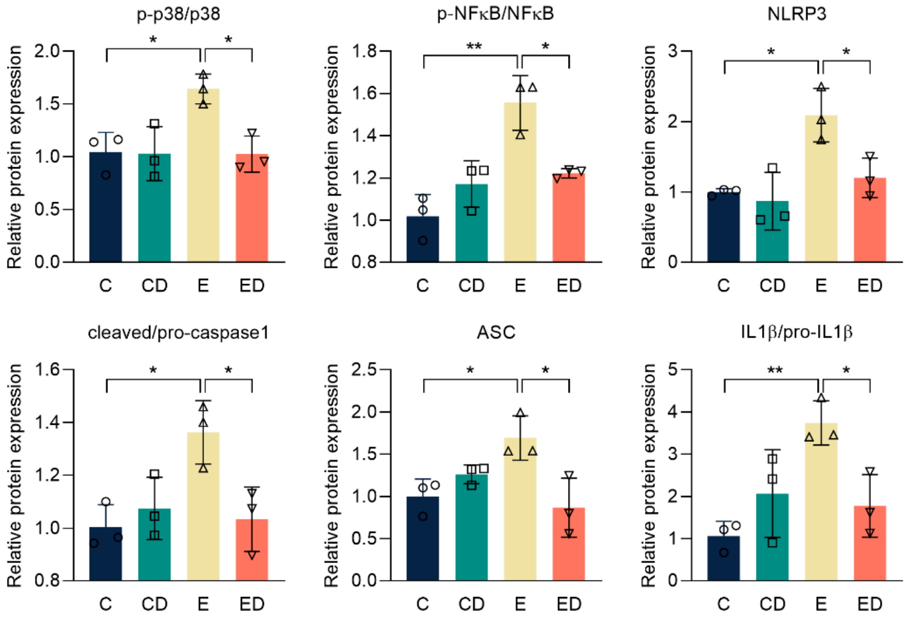

Figure S3. Quantitative analysis of protein in Figure 3E.

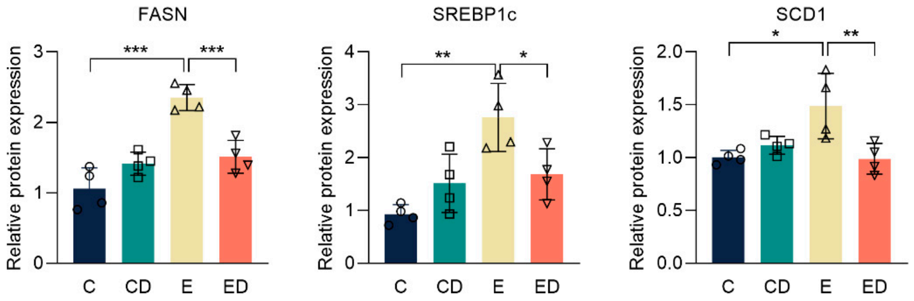

Figure S4. Quantitative analysis of protein in Figure 4B.

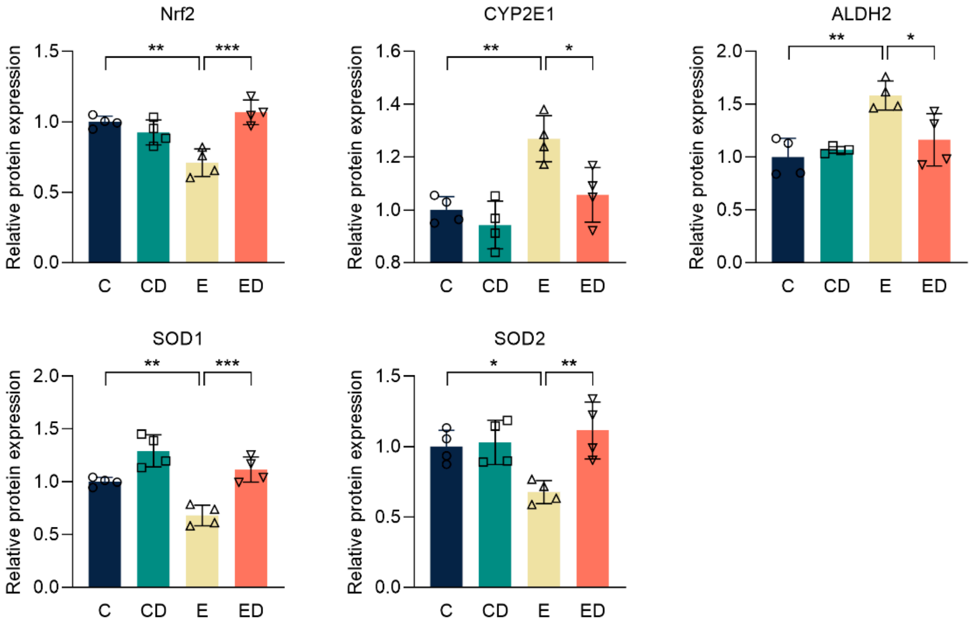

Figure S5. Quantitative analysis of protein in Figure 5C.

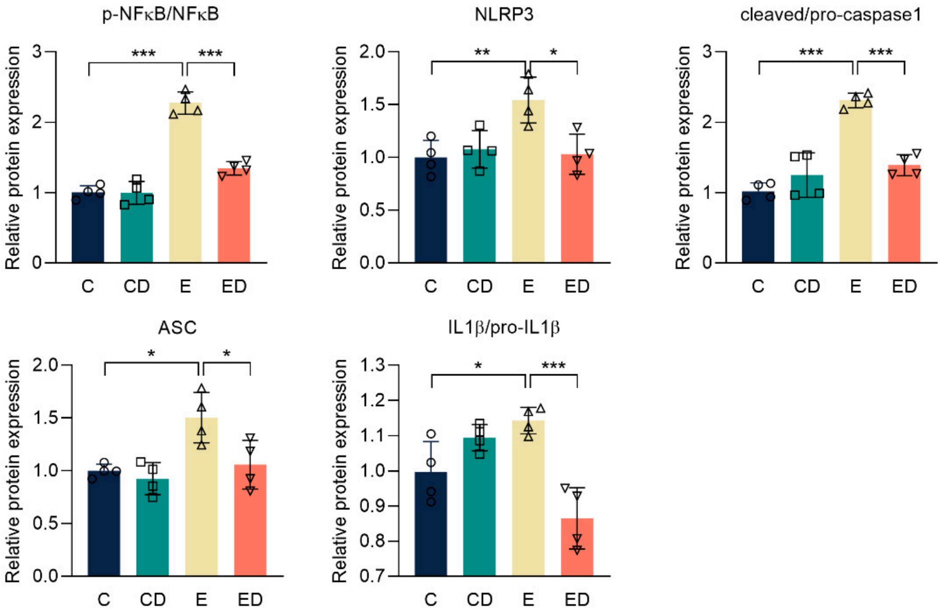

Figure S6. Quantitative analysis of protein in Figure 6A

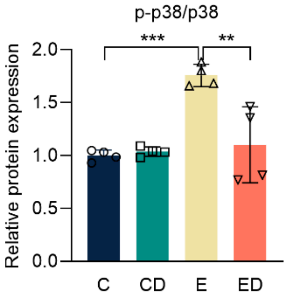

Figure S7. Quantitative analysis of protein in Figure 7A.

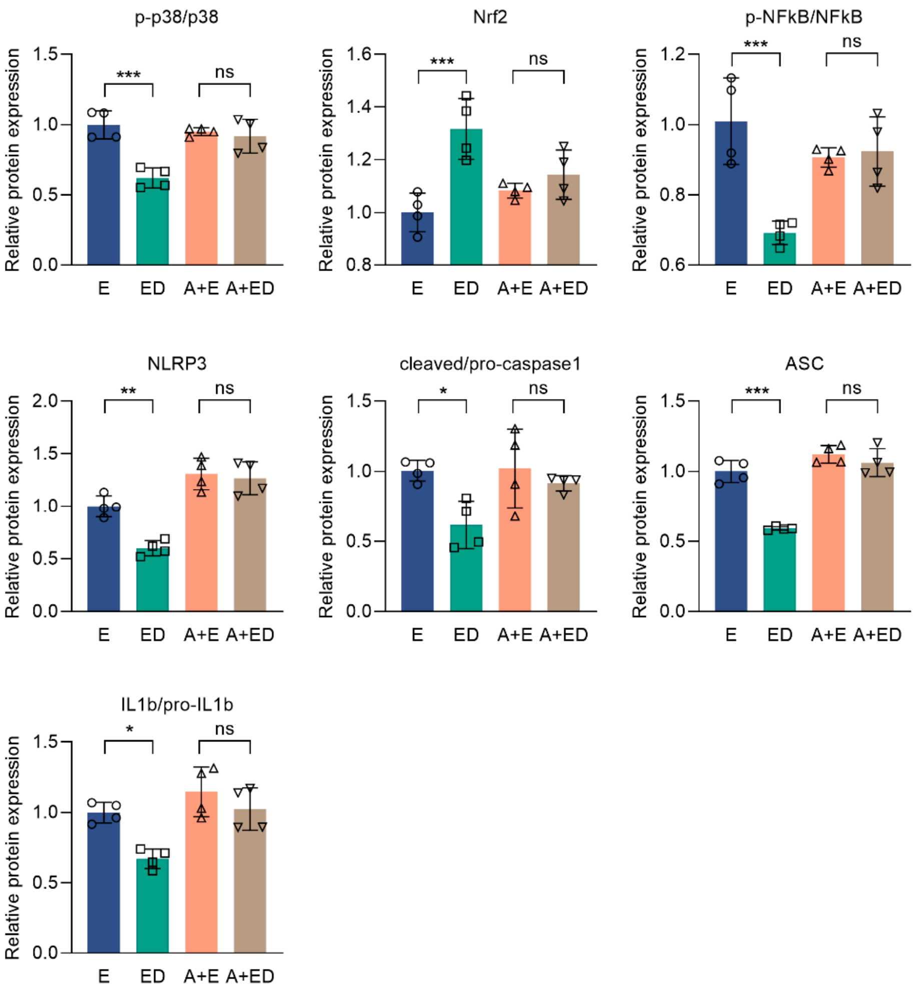

Figure S8. Quantitative analysis of protein in Figure 7B.

.

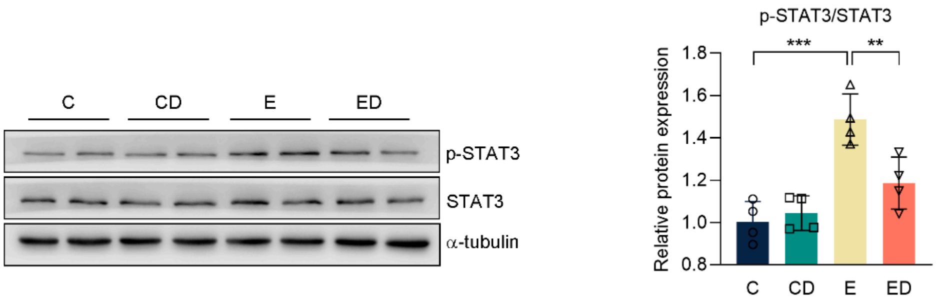

Figure S9. Protein expression of p-STAT3 and STAT3 in HepG2 cells induced by alcohol.
